# Evaluation of Anti-dsDNA Antibodies in Laboratory Practice: Management of Different Analytical Methods and Correlation with HEp-2 Immunofluorescence Patterns

**DOI:** 10.3390/antib15020023

**Published:** 2026-03-05

**Authors:** Massimo Papale, Carmela Paolillo, Loredana Iafelice, Tiziana Trivisano, Giuseppe Stefano Netti, Elena Ranieri, Gaetano Corso

**Affiliations:** 1 Laboratory of Clinical Pathology, Department of Laboratory Diagnostics, Polyclinic University-Hospital Foggia, 71122 Foggia, Italy; 2 Clinical Biochemistry, Department of Clinical and Experimental Medicine, University of Foggia, 71122 Foggia, Italy; 3 Unit of Clinical Pathology, Center for Research and Innovation in Medicine (CREATE), Department of Medical and Surgical Sciences, University of Foggia, Viale Luigi Pinto, 71122 Foggia, Italy

**Keywords:** anti-dsDNA antibody, systemic lupus erythematous, FEIA, immunoblot, CLIFT, indirect immunofluorescence

## Abstract

Background: Anti-double-stranded DNA (anti-dsDNA) antibodies are a key serological marker for systemic lupus erythematosus (SLE) and are commonly assessed in conjunction with anti-nuclear antibody (ANA) testing by indirect immunofluorescence (IIF) on HEp-2 cells. However, their detection is influenced both by the heterogeneity of the autoimmune response and by the characteristics of the analytical method employed, thereby complicating diagnostic interpretation. Methods: In this retrospective single-center study, 3090 consecutive patients undergoing anti-dsDNA analysis were screened, and 138 positive individuals, with anti-dsDNA levels ≥ 15 IU/mL by fluoroenzyme immunoassay (FEIA), were included in the study. A control group of 29 anti-dsDNA-negative patients was also analyzed. Anti-dsDNA-positive patients were stratified by antibody level (low, mild, high), and the results were correlated with HEp-2 IIF titers and fluorescence patterns. Furthermore, in a subset of 30 positive patients, anti-dsDNA antibodies were evaluated using immunoblotting (IB) and the *Crithidia luciliae* indirect immunofluorescence test (CLIFT). Statistical analyses assessed associations and concordance among methods. Results: Higher anti-dsDNA levels were generally associated with higher HEp-2 IIF titers. However, a considerable percentage (35%) of patients with positive anti-dsDNA were negative by HEp-2 IIF. Notably, high anti-dsDNA levels were detected in 19% of HEp-2 IIF-negative patients (titer < 1:80), 18% of mildly HEp-2 IIF-positive patients (titer 1:80–1:160), and 25% of HEp-2 IIF-positive patients (titer > 1:320). In the subset of 30 positive patients, FEIA analysis showed high concordance with the immunoblot in both IIF-positive (81%) and -negative (100%) patients, while CLIFT demonstrated lower agreement with both FEIA and IB independently of the IIF. Conclusions: Our findings indicate that anti-dsDNA antibody detection may occur independently of HEp-2 IIF positivity and that FEIA, especially when confirmed by immunoblot, represents a reliable approach for anti-dsDNA assessment. The observed results in this study likely reflect differences in epitope recognition and assay sensitivity among methods, suggesting the use of a multi-step diagnostic strategy in the serological evaluation of SLE.

## 1. Introduction

The presence of anti-nuclear antibodies (ANAs) is one of the immunological parameters included in the classification criteria for systemic lupus erythematosus (SLE), according to both the American College of Rheumatology (ACR) and the Systemic Lupus International Collaborating Clinics (SLICC) [1,2]. Among the various ANA specificities, anti-Sm, anti-Rib-P, and anti-dsDNA antibodies are considered highly specific for SLE. Several studies have investigated the potential of high-avidity anti-dsDNA antibodies in predicting disease onset [3], assessing disease activity and organ damage—particularly renal involvement [4]—and monitoring the treatment response [5]. However, anti-dsDNA antibodies exhibit significant heterogeneity due to the polyclonal and diverse nature of the autoimmune response to native DNA in affected patients [6], a factor that may affect the reliability of the assays used for their detection. Moreover, the presence of anti-dsDNA antibodies is not exclusive to SLE. They can be detected also in other unrelated autoimmune diseases, during infections, after treatment with biologic agents for inflammatory arthritis, and, albeit rarely, in healthy individuals [7]. Therefore, it is essential to confirm a positive anti-dsDNA result with a highly specific test, especially when these antibodies are being used for diagnostic purposes in SLE. Although the Farr assay is still considered the reference method for identifying high-avidity anti-dsDNA antibodies [8], issues related to the use of radioactive substrates have prompted the adoption of alternative non-radioactive methods, such as CLIFT, CLIA, EIA, FEIA, and MFI. Specifically, FEIA and CLIA utilize antigen–antibody interactions in the liquid phase using paramagnetic particles (microbeads), which optimally preserve the conformational structures of epitopes and improve the binding affinity [9]. Nevertheless, existing methods for anti-dsDNA antibody detection differ in avidity, affinity, antigenic specificity, and substrate type. For this reason, a stepwise diagnostic strategy has been proposed in which an initial, highly sensitive screening assay, able to detect both low- and high-affinity antibodies, is complemented by a subsequent, more specific confirmatory test [7]. CLIFT, with specificity greater than 90% compared to pathological controls, is widely recommended internationally and often used to confirm a positive result obtained through other assays. However, its low diagnostic sensitivity limits its use in primary SLE diagnosis.

Most of the studies published so far have compared CLIA and CLIFT with other methods, such as immunoblotting, the digital liquid chip method (DLCM), and ELISA. In a recent study on 91 anti-dsDNA-positive patients, Cuomo et al. [10] reported the usefulness of associating immunoblot analysis with well-established methods such as CLIA and CLIFT in order to collect more information about SLE-associated markers and facilitate diagnosis. Furthermore, Lu et al. [11] compared CLIFT, DLCM, and CLIA and showed that combining at least two assays can significantly improve the sensitivity while maintaining specificity >95%. Chang et al. [12] compared CLIA and ELISA to evaluate their diagnostic performance in terms of diagnostic power for SLE, their associations with disease activity, and their predictive value for lupus nephritis (LN). Both methods showed comparable performance for SLE diagnosis and disease activity assessment, whereas CLIA displayed greater predictive value for LN than ELISA.

**Figure 1:**
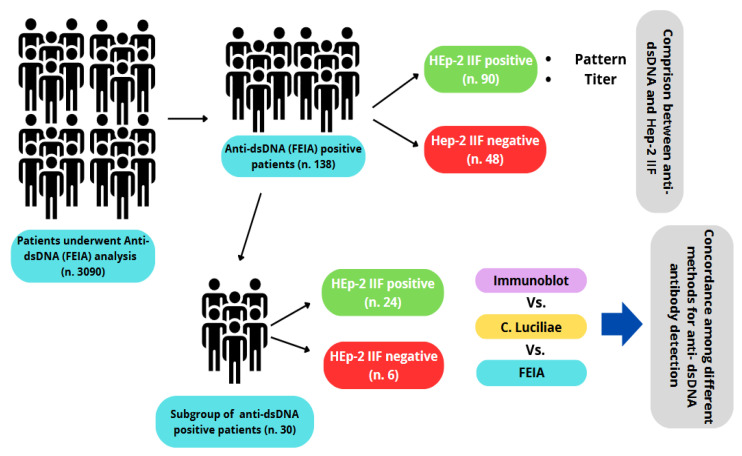
Overview of the study design.

The present study evaluated the concordance among different analytical methods for anti-dsDNA antibody measurement and their correlations with the immunofluorescence pattern in HEp-2 cells in order to establish a standardized reporting procedure, especially in cases where there is no concordance among the results between analytical methods and/or the positivity of anti-dsDNA antibodies does not correlate with the expected fluoroscopic pattern (Figure 1).

## 2. Materials and Methods

### 2.1. Patient Population Characteristics

This retrospective study was conducted at the Clinical Pathology Unit of the University Hospital “Policlinico Riuniti” of Foggia (Foggia, Italy) between May 2024 and April 2025. Written informed consent was not required since the study was conducted through a retrospective analysis of data obtained from routine laboratory tests, with the aim of comparing the results of different methods used for autoantibody characterization. All patient data were anonymized to ensure the protection of personal privacy.

The laboratory information system (WEBLAB—Tesi Group—Milan, Italy) was queried to identify all patients for whom anti-double-stranded DNA (anti-dsDNA) antibody testing had been ordered during the study period. Overall, 3090 consecutive patients were identified, of whom 138 had anti-dsDNA antibody concentrations ≥ 15 IU/mL, as determined by fluoroenzyme immunoassay (FEIA). A comparison group consisting of 29 patients with anti-dsDNA levels ≤ 14 IU/mL was selected and classified as the negative control cohort. For all patients included in the analysis, indirect immunofluorescence (IIF) patterns on HEp-2 cells were collected based on independent evaluation by two experienced pathologists. Furthermore, anti-dsDNA antibodies were reassessed in a subset of 30 patients using FEIA orthogonal methodologies—specifically, the *Crithidia luciliae* indirect immunofluorescence test (CLIFT) and immunoblotting (IB). Patient age and sex were analyzed in aggregate to assess associations with autoantibody titers. Study design is shown in Figure 1. 

### 2.2. Fluoroenzyme Immunoassay (FEIA)

Quantitative serum measurement of anti-double-stranded DNA (anti-dsDNA) antibodies was performed using the EliA™ dsDNA assayon the fully automated Phadia™ 250 platform (ThermoFisher Scientific, Waltham, MA, USA), which standardizes and automates all analytical steps. Calibration was performed using the first international standard (coded Wo/80) for anti-double-stranded DNA (anti-dsDNA). The assay showed an analytical range of 0.6–379 IU/mL, with a limit of quantification (LOQ) of 1.5 IU/mL and a limit of detection (LOD of 0.6 IU/mL.

Serum samples were diluted as required and dispensed into microplate wells coated with plasmidic double-stranded DNA, enabling the specific capture of anti-dsDNA antibodies. Following several wash steps to remove unbound antibodies, a human anti-IgG secondary antibody conjugated with a fluorescent label was added, along with the appropriate substrate. The resulting fluorescence signal, directly proportional to the concentration of anti-dsDNA antibodies, was then recorded. Each analytical run included calibration curves and/or internal quality control materials.

Results were reported as international units per milliliter (IU/mL), and anti-dsDNA levels ≤ 14 IU/mL were considered negative and ≥15 IU/mL as positive.

### 2.3. Indirect Immunofluorescence (IIF)

Anti-nuclear antibodies (ANAs) and anti-dsDNA antibodies were assessed using indirect immunofluorescence (IIF) on HEp-2 cells and *Crithidia luciliae* substrates, respectively. Sample preparation and analysis were performed using the HELIOS automated system (AESKU Diagnostics, Wendelsheim, Germany). HEp-2 pre-coated slides were used to assess nuclear and cytoplasmic fluorescence patterns, whereas *Crithidia luciliae* slides were used to detect anti-dsDNA antibodies through visualization *kinetoplast* fluorescence. Serum sample dilution, incubation, washing, FITC-conjugated anti-human IgG addition, and slide mounting were performed automatically by the HELIOS system, in accordance with the manufacturer’s instructions. Fluorescence images were acquired using an integrated high-resolution microscope. Data were analyzed using the HELIOS software (v. 4.1), which captured three distinct fields per well and determined sample positivity based on laboratory-validated cut-off values. For HEp-2 IIF, samples exhibiting positive screening fluorescence at ≥1:80 were further titrated in serial dilutions (up to 1:1280) to determine endpoint titers. For *Crithidia luciliae* IIF, anti-dsDNA positivity was defined by the specific fluorescence of the *kinetoplast*, a structure rich in double-stranded DNA. A screening titer of ≥1:10 was used to define positive samples. Only samples showing the characteristic double-spot pattern in the presence of anti-dsDNA were identified as positive. All results were independently reviewed by two experienced pathologists. According to the manufacturer’s internal data, the agreement between the AESKU HEp-2 test and the assay from another manufacturer was 100% for positive (95th % CI: 96.8–100%) and negative results (95th % CI: 85.1–100%), respectively. An analysis of 775 SLE and non-SLE patients via the AESKU nDNA assay (CLIFT) showed 42.2% sensitivity and 97.6% specificity for SLE.

### 2.4. Immunoblot Analysis

Serum samples from 30 patients were analyzed using the AESKUBLOTS^®^ ANA-17 PRO system (AESKU Diagnostics, Wendelsheim, Germany), which employs purified or recombinant antigens immobilized on nitrocellulose membrane strips, each placed in a defined and reproducible position. Analyzed autoantigens included anti-dsDNA, anti-nucleosome, anti-histone, anti-Sm, anti-U1-snRNP, anti-Ro/SSA (60 kDa and 52 kDa), anti-La/SSB, anti-Scl-70, anti-CENP-B, anti-PM-Scl, anti-Jo-1, anti-Mi-2, anti-Ku, anti-ribosomal P0, anti-PCNA, and anti-AMA-M2. The immunoblot procedure was performed using the HELIA instrument (AESKU SYSTEMS, Wendelsheim, Germany). Samples were diluted and processed according to the manufacturer’s instructions.

Briefly, each strip was put in the incubation tray and incubated with 300 μL of sample buffer diluted with 700 μL of wash buffer; subsequently, 10 μL of serum was added to each strip. After 30 min of incubation at room temperature, the strips were washed 3 times with the wash buffer, followed by the addition of diluted conjugate. Following a further 30 min of incubation at room temperature and washing steps to remove the excess conjugate, diluted substrate reagent was added to each strip and they were incubated for 15 min in the dark. Finally, after three additional washes to remove the excess substrate, antigen-specific colorimetric band intensities were evaluated by the HELIA system using its integrated camera and software. Results were classified semi-quantitatively based on the signal intensity as negative (<0.8) or positive (≥0.8). Positive results were further graded by intensity into 3 groups: 0.8–1.5 (score 1), 1.5–2.5 (score 2), and ≥2.5 (score 3). According to the manufacturer’s data, the intra- and inter-assay precision were 99.6% and 99.9%, respectively. In addition, an internal validation study including 15 sera from IIF antibody-positive patients and 50 healthy donors showed overall agreement of 98.8% (99.1% positive and 98% negative agreement, respectively).

### 2.5. Data Processing and Statistical Analysis

Data processing and statistical analysis were conducted using R (v.4.2) and IBM SPSS Statistics (v.26). Continuous variables were summarized as the mean ± standard deviation (SD). Group comparisons for continuous variables were performed using one-way analysis of variance (ANOVA). Categorical variables were compared using contingency tables and Pearson’s chi-squared test. Analytical concordance between methods was assessed by calculating the percentage agreement. Statistical significance was set at *p* < 0.05.

## 3. Results

### 3.1. Anti-dsDNA Antibody Levels

The 138 patients who tested positive for anti-dsDNA antibodies were stratified into three subgroups according to the antibody level as follow: low (15–25 IU/mL), mild (26–49 IU/mL), and high positive (>50 IU/mL). Sixty-three patients (45.6%) exhibited low anti-dsDNA levels, while 46 (33%) and 29 (21%) showed mild and high levels, respectively. Twenty-nine patients testing negative for anti-dsDNA antibodies (≤14 IU/mL) were selected as a control group. The mean ages were 59.7 ± 14.4 years and 54.8 ± 16.6 years in the negative and positive anti-dsDNA groups, respectively. When stratified into subgroups (Table 1), patients with higher anti-dsDNA antibody levels tended to be younger, although the differences regarding the other groups were not statistically significant (ANOVA, *p* = 0.105). Overall, a high female-to-male ratio was observed across all groups, except the highpositive group, which had a more balanced ratio (1.07:1).

### 3.2. Correlation Between HEp-2 Fluorescence Titers and Plasma Levels of Anti-dsDNA Antibodies

**Figure 2:**
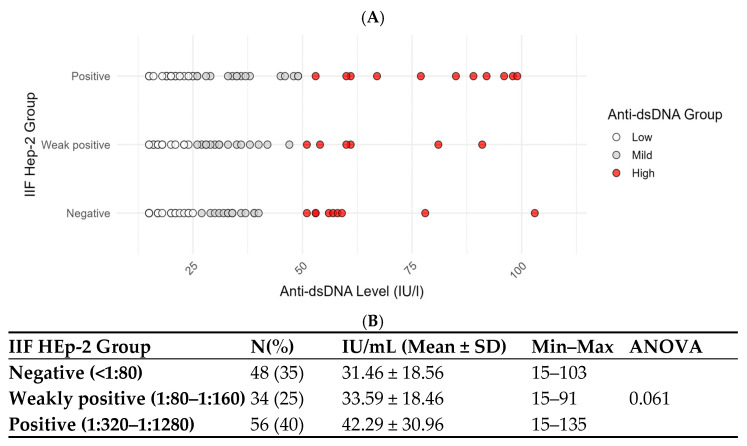
Anti-dsDNA levels stratified by HEp-2 IIF positivity. (**A**) Anti-dsDNA groups: low = 15–25 IU/mL (white dots); mild = 26–49 IU/mL (grey dots); high= over 50 IU/mL (red dots). HEp-2 IIF groups: negative ≤ 1:80; weaklypositive = 1:80–1:160; positive = 1:320–1:1280. (**B**) Anti-dsDNA mean level distribution across HEp-2 IIFgroups. The differences did not reach statistical significance (ANOVA, *p* = 0.061), although a trend toward higher anti-dsDNA levels with increasing HEp-2 IIF titers was observed.

To better understand the relationship between fluorescence patterns in HEp-2 cells and anti-dsDNA antibody levels, we analyzed the HEp-2 IIF titers in the 138 anti-dsDNA-positive patients by FEIA. Correlation analysis was performed, regardless of the pattern type. Based on the HEp-2 IIF results, patients were divided into three groups: negative (titers < 1:80), weakly positive (titers from 1:80 to 1:160), and positive (titers from 1:320 to 1:1280). Anti-dsDNA levels increased progressively across subgroups, with mean values of 31.46 ± 18.56 IU/mL in the IIF negative group, 33.59 ± 18.46 IU/mL in the weakly positive group, and 42.29 ± 30.96 IU/mL in the positive group (Figure 2). Moreover, we cross-stratified the 138 patients according totheir HEp-2 IIF titers (negative, weakly positive, and positive) and anti-dsDNA levels (low, mild, and high positive groups). The data reported in Table 2 show that, among the HEp-2 IIF-negative group, 24 patients (50%) had low anti-dsDNA levels, 15 (31%) had mild levels, and 9 (19%) had high levels. This indicates that, even among patients with a negative IIF HEp-2 titer, a notable proportion still had positive anti-dsDNA antibodies, including 19% with high levels. In the IIF HEp-2 weakly positive group, 13 patients (38%) showed low anti-dsDNA levels, 15 (44%) had mild levels, and 6 (18%) had high levels. In the IIF HEp-2-positive group, 26 patients (46%) had low levels, 16 (29%) had mild levels, and 14 (25%) had high levels of anti-dsDNA antibodies. Chi-squared analysis indicated no statistically significant association between IIF HEp-2 titers and anti-dsDNA levels (Pearson χ^2^ = 2.97, *p* = 0.563).

### 3.3. Correlation Between Anti-dsDNA Antibodies and HEp-2 IIFPatterns

**Figure 3:**
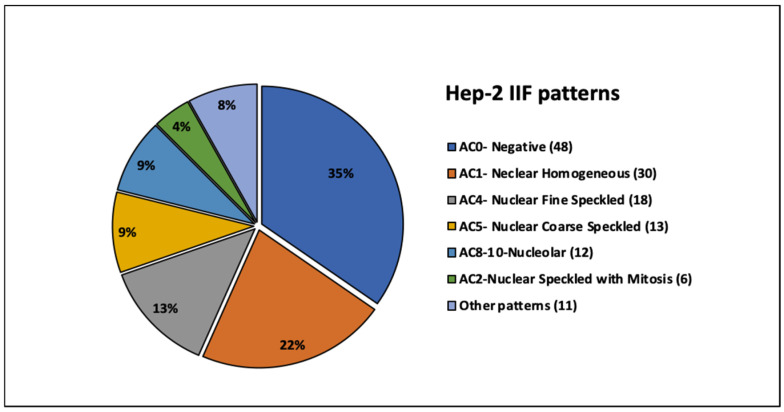
HEp-2 IIF patterns across anti-dsDNA-positive patients. Distribution of HEp-2 IIF patterns within selected patients. The percentage of cases found for each type of IIF pattern is shown in the pie chart, while the number of samples is shown in brackets.

In order to explore potential correlations between anti-dsDNA antibodies and HEp-2 IIF patterns, the antibody results were compared with the fluoroscopic profiles. Forty-eight anti-dsDNA-positive patients (35%) were negative by HEp-2 IIF, while 65% were IIF-positive. Among positive patients, the following main patterns were observed: homogeneous (22%), fine speckled (13%), coarse speckled (9%), nucleolar (9%), and speckled with positive mitoses (4%). Less frequent patterns, recognized in 8% of cases, were classified as other patterns. They included AC3—centromeric (N = 3), AC29—DNA topoisomerase I-like (N = 2), AC8-10—nuclear dots (N = 2), AC27—intracellular bridges (N = 1), AC18—cytoplasmic GW bodies (N = 1), AC22—cytoplasmic Golgi-like (N = 1), and AC21—AMA-like mitochondrial staining (N = 1), according to the International Consensus on ANA Patterns (ICAP) (Figure 3).

### 3.4. Comparison of Positivity Rates Across Different Anti-dsDNA Detection Methods

To compare how well different methods may detect anti-dsDNA antibodies, we analyzed a panel of 30 serum samples using several complementary assays. Not all methods were applied to the full set of samples, depending on sample availability (Table 3 and Table S1). Since patients were selected based on the result of the FEIA analysis (28 positive and 2 negative), the other methods were tested for their ability to confirm positive or negative cases detected by FEIA. In total, 73% of FEIA-positive patients had positive nuclear patterns on HEp-2 cells, while 21% were HEp-2-negative. Furthermore, 79% and 24% of FEIA-positive patients showed positive anti-dsDNA antibodies when measured by immunoblot and CLIFT, respectively. Finally, antibodies against related nuclear antigens were less common, with a 10% positivity rate for the nucleosome and none for the histone (Table 3 and Table S1). When compared with each other, FEIA and immunoblot showed consistent results in 86% of cases, while CLIFT agreed with the other methods in 32% of cases. A comparative analysis, in patients divided according to the nuclear pattern, showed 81% and 100% agreement between FEIA and immunoblot in patients with positive and negative HEp-2 IIF patterns, respectively. Finally, the agreement between CLIFT and the other two methods was 40% in IIF-negative patients and 30% in IIF-positive patients (Table 3B and Table S1).

## 4. Discussion

The present study investigated, in a cohort of 138 patients testing positive for anti-double-stranded DNA (anti-dsDNA) antibodies, the relationship between this biomarker and the results of an indirect immunofluorescence (IIF) analysis of HEp-2 cells, as well as the concordance amongresults across different analytical techniques for anti-dsDNA antibody detection. Given the well-established association between anti-dsDNA antibody titers and disease activity or flares, patients were stratified into three subgroups based on their antibody levels—low (15–25 IU/mL), medium (26–49 IU/mL), and high (>50 IU/mL)—to allow for a more refined evaluation of the relationships between anti-dsDNA levels and the other parameters examined. In this cohort, 79% of patients exhibited low or medium anti-dsDNA titers, whereas the remaining 21% showed high titers. Although systemic lupus erythematosus (SLE) is known to be more prevalent in females [13,14], the patients with higher antibody levels displayed a more balanced male-to-female ratio. These findings may suggest that disease activity and severity—often associated with elevated anti-dsDNA titers [15,16,17]—may not be strongly influenced by sex. Furthermore, in HEp-2 IIF-positive patients, anti-dsDNA antibody levels tended to correlate with fluorescence titers, and, in accordance with the literature, the main observed patterns were homogeneous, speckled, and nucleolar. Notably, a substantial proportion of anti-dsDNA-positive patients were, surprisingly, negative inHEp-2 IIF. Recent studies have reported this pattern in both SLE [18] and non-SLE cohorts [19], although other works [20] consider it a rare and uncommon event. In our cohort, 50% of HEp-2 IIF-negative patients had low anti-dsDNA levels, whereas the remaining ones showed moderate (31.25%) to high (18.75%) anti-dsDNA antibody levels—a pattern that could paradoxically suggest a more severe disease phenotype.

Positivity foranti-dsDNA antibodies, particularly in patients with low titers, may depend on several factors, including the high analytical sensitivity of the fluorescence enzyme immunoassay (FEIA), which is considered one of the most reliable methods for the quantification of these autoantibodies.

In this regard, a recent meta-analysis [21] evaluated the specificity of different methods for anti-dsDNA antibody detection across 30 studies published between 2004 and 2019, in which true positives, true negatives, false positives, and false negatives were clearly defined for each assay. FEIA, together with the *Crithidia luciliae* indirect immunofluorescence test (CLIFT), demonstrated the highest specificity (>90%) for systemic lupus erythematosus (SLE). In contrast, for other methodologies, such as the enzyme immunoassay (EIA) and chemiluminescent immunoassay (CLIA), the available data were insufficient to determine whether the specificity exceeded the 90% threshold recommended in thecurrent guidelines. These findings support the high analytical reliability of anti-dsDNA results obtained by FEIA. Nevertheless, even with such a sensitive and specific method, an estimated 5–8% of cases may represent falsepositive results. Overall, the observed discordance between FEIA-positive and HEp-2 indirect immunofluorescence (IIF)-negative samples likely reflects the greater analytical sensitivity of FEIA compared with IIF, as well as differences in epitope recognition between the two methodologies; less frequently, the discrepancy may be attributable to falsepositive results.

To further investigate these hypotheses, anti-dsDNA antibody testing was repeated in a subgroup of 30 patients using immunoblotting and indirect immunofluorescence on *Crithidia luciliae*. In this subgroup, higher concordance was observed between FEIA and immunoblotting (86%), whereas the concordance between CLIFT and the other methods was lower (32%), as previously reported [22]. This observation reinforces the hypothesis that different assays recognize distinct epitopes [18,23], some of which may not be adequately represented or accessible in the HEp-2 IIF substrate despite being present in patients’ sera. Importantly, in HEp-2 IIF-negative patients retested using alternative methods, complete concordance was observed between FEIA and immunoblotting. Moreover, two IIF-negative patients were positive for anti-nucleosome antibodies, which have been reported to be a more sensitive diagnostic marker for SLE than anti-dsDNA antibodies [24]. These results may further support the diagnostic reliability of anti-dsDNA antibody positivity in SLE, even in the absence of HEp-2 IIF reactivity.

Several limitations of this study should be acknowledged, including its single-center design and the relatively limited sample size. Moreover, the lack of detailed clinical information prevented the correlation of laboratory findings with patients’ clinical status, such as newly diagnosed disease versus disease flares, as well as with ongoing therapies. While it is evident that future studies including larger and well-characterized cohorts will be necessary to address these issues, the present findings highlight that, in the absence of general guidelines, each case should be carefully evaluated by the clinician to ensure an interpretation that is more consistent with the patient’s clinical condition.

## 5. Conclusions

This study provides practical laboratory guidance for the management of anti-dsDNA antibody positivity, particularly in patients with negative HEp-2 IIF results. In this cohort, FEIA-positive anti-dsDNA findings warrant re-evaluation using alternative methods, and laboratory reports should include a comment on the concordance or discordance among the different assays. Such an approach may be especially valuable in cases of discrepant results, allowing clinicians to consider longitudinal retesting when a falsepositive result is suspected.

## Figures and Tables

**Table 1:** Cross-comparison of anti-dsDNA levels and patient age.

Anti-dsDNAGroup	N	Anti-dsDNA (IU/mL)	Age Mean ± SD
Negative (≤14 IU/mL)	29	8.6 ± 2.7	59.4 ± 14.9
Low (15–25 IU/mL)	63	19.1 ± 3.4	57.8 ± 17.4
Mild (26–49 IU/mL)	46	34.8 ± 6.4	53.3 ± 16.4
High (≥50 IU/mL)	29	76.3 ± 23.7	50.8 ± 14.1
ANOVA			*p* = 0.105

**Table 2:** Distribution of numbers (N) of patients according to HEp-2 IIF and anti-dsDNA groups. No statistically significant differences were observed across subgroups (chi-squared Pearson = 2.9695, *p* = 0.5629).

HEp-2 IIF Group	Total	Anti-dsDNA Low	Anti-dsDNA Mild	Anti-dsDNA High
		(15–25 IU/mL)	(26–49 IU/mL)	(over 50 IU/mL)
		N (%)	N (%)	N (%)
**Negative < 1:80**	48	24 (50)	15 (31)	9 (19)
**Weakly positive (1:80–1:160)**	34	13 (38)	15 (44)	6 (18)
**Positive (1:320–1:1280)**	56	26 (46)	16 (29)	14 (25)
**Total**	138	63 (46)	46 (33)	29 (21)

**Table 3:** Cross-comparison among complementary methods. (**A**) Prevalence of positive nuclear pattern and anti-dsDNA antibodies tested by FEIA, immunoblot, and CLIFT in an independent set of 30 samples. Immunoblot results for anti-dsDNA-associated antibodies (nucleosome and histones) are also reported. (**B**) Percentage of agreement across different anti-dsDNA detection methods in HEp-2-positive and -negative groups.

(**A**)				
**Marker**	**N**	**Positive (%)**	**Negative (%)**	**NA**
**Cell pattern (IIF-HEp-2)**	30	22 (73)	8 (27)	--
**dsDNA (FEIA)**	30	28 (93)	2 (7)	--
**dsDNA (Immunoblot)**	29	23 (79)	6 (21)	1
**dsDNA (CLIFT)**	25	6 (24)	19 (76)	5
**Nucleosome (Immunoblot)**	29	3 (10.3)	26 (89.7)	1
**Histone (Immunoblot)**	29	0 (0)	100 (100)	1
(**B**)				
**Anti-dsDNA positivity concordance (%)**				
		**FEIA vs. IB (agreement %)**	**FEIA vs. CLIFT (agreement %)**	**IB vs. CLIFT**
				**(agreement %)**
**IIF-HEp-2 negative**		100	40	40
**IIF-HEp-2 positive**		81	30	30
**IIF-HEp-2 (all)**		86	32	32

## Data Availability

No new data were created for this study. Patient data are unavailable due to patient privacy.

## Supplementary Materials

The following supporting information can be downloaded at https://www.mdpi.com/article/10.3390/antib15020023/s1. Table S1: Results of anti-dsDNA antibody analysis by FEIA, CLIFT, and IB.
