# Supplementary material for: Evaluation of Anti-dsDNA Antibodies in Laboratory Practice: Management of Different Analytical Methods and Correlation with HEp-2 Immunofluorescence Patterns
Source: Antibodies (Basel). 2026 Mar 5;15(2):23. doi: 10.3390/antib15020023 (PMC13010703; doi:10.3390/antib15020023)
Supplement: Supplementary file 1 [file antibodies-15-00023-s001.zip › antibodies-4137757-supplementary.pdf]

	Anti-dsDNA			ds-DNA associated autoantigens		IIF-Hep-2	
**ID**	**FEIA (UI/mL)**	**CLIFT**	**IB**	**Necleosome**	**Histone**	**Pattern**	**Titer**
1345057	0,5	Negative	0	Negative	Negative	AC1- Nuclear Homogeneus	1280
4214567	10	Negative	0	Negative	Negative	AC4- Nuclear Fine Speckled	160
4828035	15	Negative	3	Negative	Negative	Negative	0
4121244	19	Negative	3	Negative	Negative	AC22- Cytoplasmic Golgi	320
4083410	20	n.a	2	Negative	Negative	AC1- Nuclear Homogeneus	1280
4029036	24	Negative	1	Negative	Negative	AC1- Nuclear Homogeneus	1280
1203758	25	Negative	2	Negative	Negative	AC1- Nuclear Homogeneus	640
1190633	28	Negative	1	Negative	Negative	AC4- Nuclear Fine Speckled	640
1280712	31	Negative	0	Negative	Negative	AC7-Nuclear dots	80
**4118742**	**32**	**Positive**	**3**	**Positive**	**Negative**	**Negative**	**0**
1122731	33	Negative	1	Negative	Negative	Negative	0
4076244	33	Negative	2	Negative	Negative	AC4- Nuclear Fine Speckled	160
**4121285**	**35**	**Negative**	**3**	**Positive**	**Negative**	**AC5- Nuclear Coarse Speckled**	**320**
4131649	35	Negative	1	Negative	Negative	Nucleolar	160
9012863	36	Negative	1	Negative	Negative	AC4- Nuclear Fine Speckled	320
4136440	46	Negative	2	Negative	Negative	AC1- Nuclear Homogeneus	640
9018290	51	**Positive**	0	Negative	Negative	AC1- Nuclear Homogeneus	120
1123595	53	Negative	2	Negative	Negative	Negative	0
4181999	53	**Positive**	2	Negative	Negative	Negative	0
4160265	54	Negative	1	Negative	Negative	AC5- Nuclear Coarse Speckled	160
**4165795**	**59**	**n.a.**	**2**	**Positive**	**Negative**	**Negative**	**0**
1075046	60	Negative	0	Negative	Negative	AC1- Nuclear Homogeneus	640
4157889	60	Negative	2	Negative	Negative	AC1- Nuclear Homogeneus	1280
4079838	77	**Positive**	1	Negative	Negative	AC4- Nuclear Fine Speckled	320
4155862	78	n.a.	1	Negative	Negative	Negative	0
1085903	81	n.a.	n.a.	n.a.	n.a.	AC1- Nuclear Homogeneus	160
4089070	91	Positive	0	Negative	Negative	AC4- Nuclear Fine Speckled	80
4060210	98	Negative	2	Negative	Negative	AC1- Nuclear	640

						Homogeneus	
1301290	103	n.a	2	Negative	Negative	Negative	0
9034427	135	Positive	3	Negative	Negative	AC1- Nuclear Homogeneus	1280

Table S1. Results of anti-dsDNA antibody analysis by FEIA, CLIFT and IB. Anti-dsDNA associated autoantigens and IFF-Hep-2 pattern of each sample is also reported. Patients showing positivity for both anti-dsDNA and anti-nucleosome are reported in bold. Of note, samples code 4221244, 4221285, 4260265 and 1075046 were also positive for Pm-scl, cenp, scl-70 and La, respectively
